# Calcium influx through T-type calcium channels regulates secondary necrosis and inflammation through recruitment of ATG9A-mediated membrane repair

**DOI:** 10.1038/s41419-026-09070-9

**Published:** 2026-07-08

**Authors:** Omar F. Audi, Dilara C. Ozkocak, Chad Johnson, Bo Shi, Jascinta P. Santavanond, Caitlin L. Vella, Joanne M. Hildebrand, Robyn Sharples, Quan Thinh Le, Lesley Cheng Sim, Amy A. Baxter, Mark D. Hulett, Ivan K. H. Poon, Thanh Kha Phan

**Affiliations:** 1 Department of Biochemistry and Chemistry, La Trobe Institute for Molecular Science, La Trobe University, Melbourne, Bundoora, VIC 3086 Australia; 2 Research Centre for Extracellular Vesicles, La Trobe Institute for Molecular Science, La Trobe University, Melbourne, VIC 3086 Australia; 3 The Walter and Eliza Hall Institute, Parkville, VIC 3052 Australia

**Keywords:** Apoptosis, Cell signalling

## Abstract

Approximately 200–300 billion cells die daily through apoptosis, a prominent form of programmed cell death, to maintain tissue homoeostasis. If apoptotic cells are not efficiently removed by phagocytes, they progress to secondary necrosis when the plasma membrane (PM) becomes permeabilised and release proinflammatory damage-associated molecular patterns (DAMPs) such as HMGB1 and ATP, which drive inflammation and contribute to autoimmune diseases. Thus, controlling inflammation through maintaining PM integrity is critical, however the molecular mechanisms underpinning this is not well defined. Here, we reveal a calcium-dependent process that delays secondary necrosis by promoting PM repair. Mechanistically, calcium influx through T-type voltage-gated calcium channels mediates the recruitment of the lipid scramblase ATG9A and Golgi components to damaged PM regions, thereby preventing early cellular lysis and DAMP release. Inhibition of calcium influx or loss of ATG9A accelerates PM rupture, increases DAMP secretion, and exacerbates inflammatory cell recruitment in vivo. Taken together, this study establishes a novel role for T-type calcium channels and ATG9A in regulating PM repair during apoptosis and highlights their therapeutic potential for controlling unwanted inflammation.

## Introduction

To maintain tissue homoeostasis, billions of cells are turned over through apoptosis every day. Apoptotic cells are cleared by phagocytes such as macrophages and epithelial cells through a process termed ‘efferocytosis’ [1]. If phagocytes fail to eliminate cell corpses promptly, apoptosis can proceed to secondary necrosis. At this stage, dying cells undergo membrane rupture and release autoantigens as well as proinflammatory cellular contents such as danger-associated molecular patterns (DAMPs). DAMPs, particularly nuclear protein HMGB1 and cytoplasmic ATP, have been well-documented in promoting immune cell infiltration and ultimately the onset of inflammation [2]. Therefore, defects in efferocytosis and/or the persistence of DAMPs underpin the pathogenesis of many chronic inflammatory and autoimmune conditions such as arthritis, systemic lupus erythematosus and Sjogren’s disease [2].

Upon PM damage, cells can employ a variety of mechanisms to promote membrane repair [3]. A major signal to initiate membrane repair is the influx of calcium (Ca^2+^) ions from the extracellular space, signalling for membrane patching by recruiting organelle materials or removal of damaged areas through membrane budding [3, 4]. In the context of cell death, recent work has demonstrated a calcium influx-dependent membrane repair process for lytic cell death modalities such as pyroptosis and necroptosis [5]. During pyroptotic and necroptotic cell death, calcium influxes into the cytoplasm, through membrane damages caused by oligomerisation of pore-forming protein Ninjurin-1 (NINJ1) and caspase 3-activated GSDME [6, 7]. Subsequently, this triggers the translocation of Golgi-resident lipid scramblase autophagy-related protein 9A (ATG9A) to the plasma membrane, where it orchestrates membrane repair through engaging with ESCRT machinery [5]. Notably, whether this calcium-influx repair mechanism is activated during apoptosis to limit progression to secondary necrosis is yet to be determined.

Our previous work observed the influx of calcium through low voltage-gated T-type calcium channels during apoptosis, in particular channel isoforms Ca_V_3.1 and 3.2 [8]. Thus, in this study we investigate the role of calcium influx through T-type calcium channels and ATG9A in the context of membrane repair during the transition of apoptosis to secondary necrosis. We report that preventing calcium influx through isoforms 3.1 and 3.2 can hasten the onset of secondary necrosis. Notably, we determined that ATG9A and Golgi components are recruited to the PM to prevent damages, release of DAMPs, and immune cell recruitment. Our findings suggest that regulated calcium entry during apoptosis is important to maintain membrane integrity by preventing uncontrolled DAMP release.

## Results

### Calcium influx during apoptosis regulates plasma membrane rupture and the onset of secondary necrosis

**Fig. 1:**
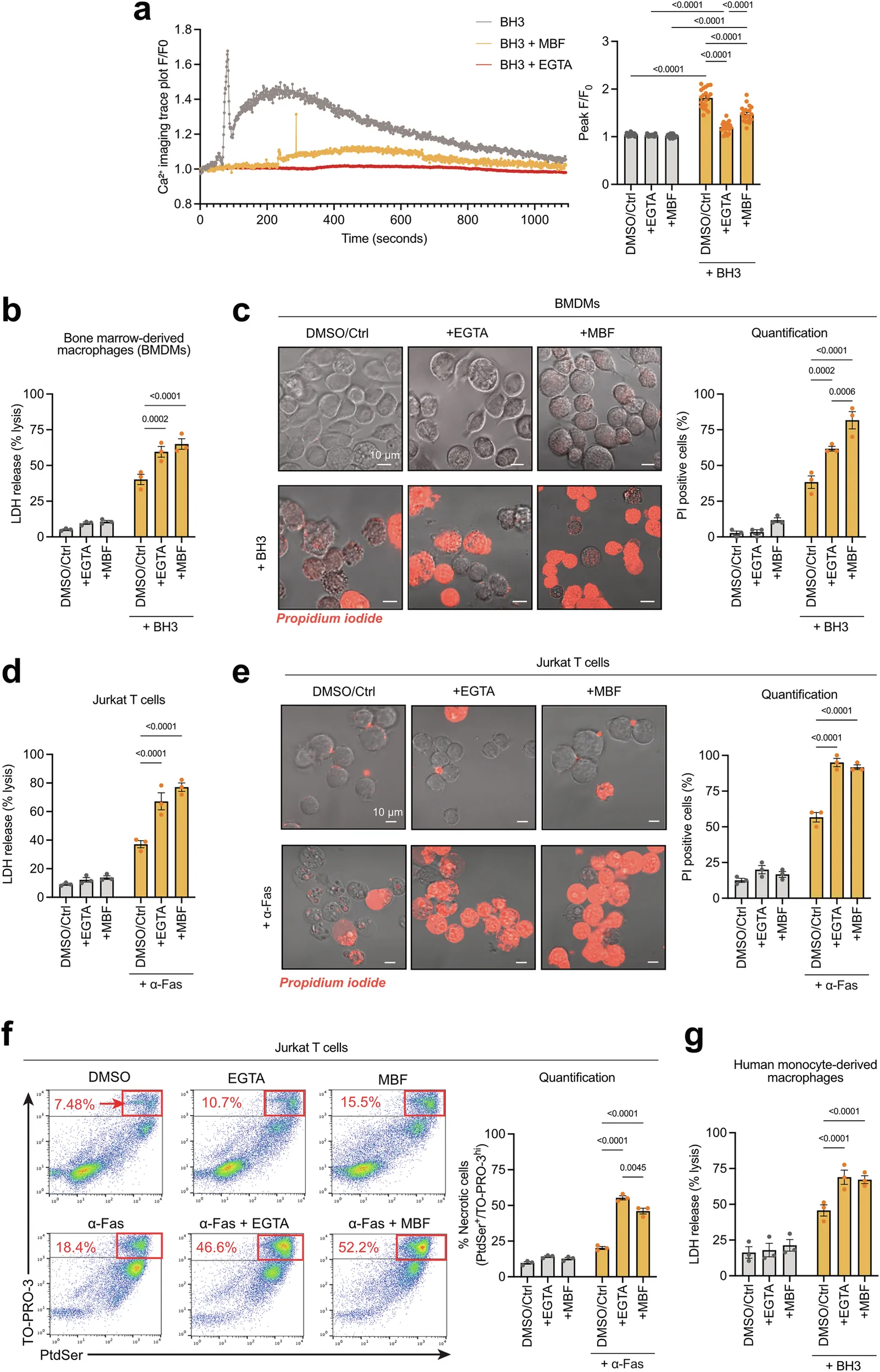
The onset of secondary necrosis is regulated by calcium influx through T-type calcium channels during apoptosis. **a** Fluo-4 fluorescence intensity (F/F_0_) measured by time-lapse microscopy-based calcium imaging, in WT BMDMs following apoptosis induction and Ca^2+^ influx blockade with EGTA (1 mM) and MBF (10 µM). **b**, **c** Quantification of membrane lysis in WT BMDMs at 10 h post-apoptosis induction by LDH release assay and PI uptake assay, respectively. **d**–**f** Quantification of membrane lysis in Jurkat T cells at 10 h post-apoptosis induction by LDH release assay, PI uptake assay and flow cytometry analysis, respectively. **g** Quantification of membrane lysis in human monocyte-derived macrophages (hMDMs) at 10 h post-apoptosis induction by LDH release assay. Data represent three independent experiments (*n* = 3). Error bars represent s.e.m. Statistical analyses were conducted using two-way ANOVA with Tukey’s post hoc test to identify significant differences among treatment groups, *p < 0.05, **p < 0.01, ***p < 0.001; ns not significant. Scale bar = 10 μm.

Calcium influx reportedly mediates membrane repair upon plasma membrane rupture (PMR) during lytic cell death [5]. As we previously recorded, calcium influxes mediated by T-type voltage-gated Ca^2+^ channels in various cell types undergoing apoptosis, we set to determine whether this apoptotic calcium influx regulates secondary necrosis [8]. We performed calcium imaging using Fluo-4 as a calcium probe and quantified the calcium mobilisation by peak fluorescence intensity (F/F_0_). We were able to confirm a sustained increase in cytoplasmic Ca^2+^ in BMDMs, a common cell model to study PMR, following treatment with BH3 mimetics (apoptosis stimuli) (Fig. 1a). This calcium influx was readily inhibited by EGTA (an extracellular calcium chelator) and mibefradil (MBF, a T-type calcium channel blocker [9–11]. suggesting a regulated Ca^2+^ entry from the extracellular milieu. Using a lactate dehydrogenase (LDH) release assay, a proxy measure of PMR, we found that EGTA and MBF significantly exacerbated PMR in apoptotic BMDMs (Fig. 1b). Consistently, EGTA and MBF also enhanced the uptake of propidium iodide (PI), a membrane-impermeable fluorescent dye used as PMR indicator (Fig. 1c). Apoptotic human Jurkat T cells (treated with anti-Fas, another apoptotic stimulus) (Fig. 1e, f) and BH3 mimetics-treated human monocyte-derived macrophages (Fig. 1g) showed similar increase in PMR level upon EGTA or MBF treatments according to LDH release and PI uptake via flow cytometry and confocal microscopy (Fig. 1e–g). Our data suggest calcium influx during apoptosis may play a role in regulating the onset of PMR as apoptosis progresses into a secondary necrotic state.

### T-type calcium channels mediate apoptotic calcium influx and regulate secondary necrosis

**Fig. 2:**
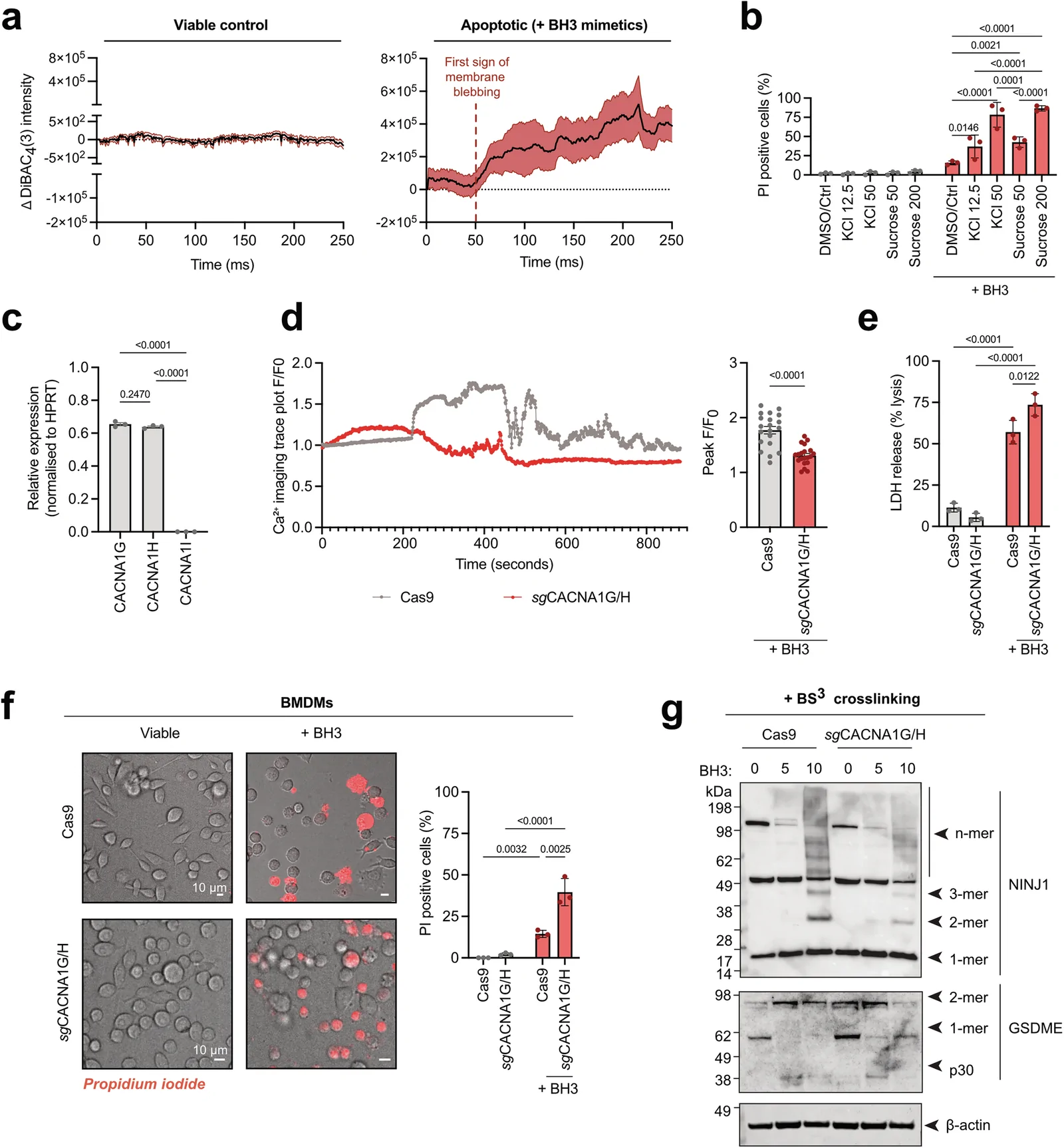
The onset of secondary necrosis is dependent on Ca_V_3.1 and Ca_V_3.2 channels. **a** Measurement of changes in DiBAC_4_(3) intensity of cells following apoptosis induction (via BH3 mimetics), compared to the viable control. The dotted line represents the first sign of apoptosis through membrane blebbing. Shaded red areas represent s.e.m. **b** Measurement of membrane lysis via PI uptake in WT BMDMs following induction of secondary necrosis and treatment with membrane potential modulators KCl (12.5 or 50 mM) or sucrose (50 and 200 mM). **c** Quantitative PCR analysis of CACNA1G, CACNA1H, and CACNA1I expression in WT BMDMs. **d** Fluo-4 fluorescence intensity (F/F_0_) measured by time-lapse microscopy-based calcium imaging, in Cas9 and *sg*CACNA1G/H BMDMs following apoptosis induction. **e**, **f** Quantification of membrane lysis in WT BMDMs at 10 h post-apoptosis induction by LDH release assay and PI uptake assay, respectively. **g** Immunoblotting of NINJ1 and GSDME oligomerisation during Ca^2+^ blockade in cells deficient in Ca_V_3.1 and Ca_V_3.2. Data represent three independent experiments (*n* = 3). Error bars represent s.e.m. Statistical analyses were conducted using two-way ANOVA with Tukey’s post hoc test (for **b**, **e**, **f**), one-way ANOVA with Dunnett’s post hoc test (for **c**), or student’s unpaired *t*-test (for **d**), to identify significant differences among treatment groups, *p < 0.05, **p < 0.01, ***p < 0.001; ns not significant. Scale bar = 10 μm.

Since the plasma membrane remains largely intact prior to secondary necrosis, the observed Ca²⁺ influx during apoptosis is likely to occur through regulated pathways, such as a change in membrane potential. Indeed, performing confocal imaging using DiBAC_4_(3), a voltage-sensitive probe that enters depolarised cells and exhibits enhanced fluorescence, we detected membrane depolarisation post-apoptosis induction (determined as the first sign of membrane blebbing) (Fig. 2a). Perturbation of membrane potential by hypertonic KCl [12] or hyperosmotic sucrose [13] also exacerbated PMR in BH3-treated BMDMs (Fig. 2b). We therefore proposed that Ca²⁺ entry during apoptosis may be regulated in a voltage-gated manner. As MBF impaired calcium influx during apoptosis and exacerbated downstream PMR, we sought to determine whether voltage-gated T-type calcium channels act as regulators of secondary necrosis. To determine which T-type channel isoform is involved in regulating secondary necrosis, we conducted qPCR in BMDMs. Among the three channel isoforms (Ca_V_3.1, Ca_V_3.2, and Ca_V_3.3, encoded by CACNA1G, CACNA1H, and CACNA1I respectively), BMDMs predominantly expressed CACNA1G and CACNA1H as shown by qPCR analysis (Fig. 2c). Using CRISPR/Cas9 gene editing, we generated CACNA1G and CACNA1H double knockout BMDMs (*sg*CACNA1G/H) (Supplementary Fig. 1a). Compared to the Cas9 control, BH3 mimetics-treated CACNA1G/H-deficient BMDMs showed significantly reduced calcium influx without changes in caspase 3–7 mediated apoptotic cell death (Fig. 2d–f and Supplementary Fig. 1c). Consistently, the CACNA1G/H-deficiency also worsened PMR in BMDMs, evidenced by increased LDH release and PI uptake (Fig. 2e, f). Of note, the observed PMR in wild-type and CACNA1G/H-deficient cells were only impaired upon caspase inhibition by QVD, but not RIP-kinase inhibition by necrostatin-1, suggesting apoptosis-induced secondary necrosis rather than necroptosis (Supplementary Fig. 1d).

Since NINJ1 and GSDME oligomerise on apoptotic BMDM membranes and promote PMR during secondary necrosis [14], we hypothesised that T-type calcium channel-mediated calcium influx would negatively regulate these lytic pore-forming proteins. However, we performed crosslinking experiments and found that perturbation of apoptotic calcium influx (by genetic disruption of T-type calcium channel) did not increase NINJ1 or GSDME oligomerisation (Fig. 2g). Our data therefore suggest that apoptosis-associated changes in membrane potential induce Ca^2+^ entry through T-type calcium channels to regulate the onset of secondary necrosis through a mechanism independently of suppression of NINJ1 and GSDME oligomerisation.

### T-type calcium channel-mediated calcium influx promotes ATG9A translocation for membrane repair

**Fig. 3:**
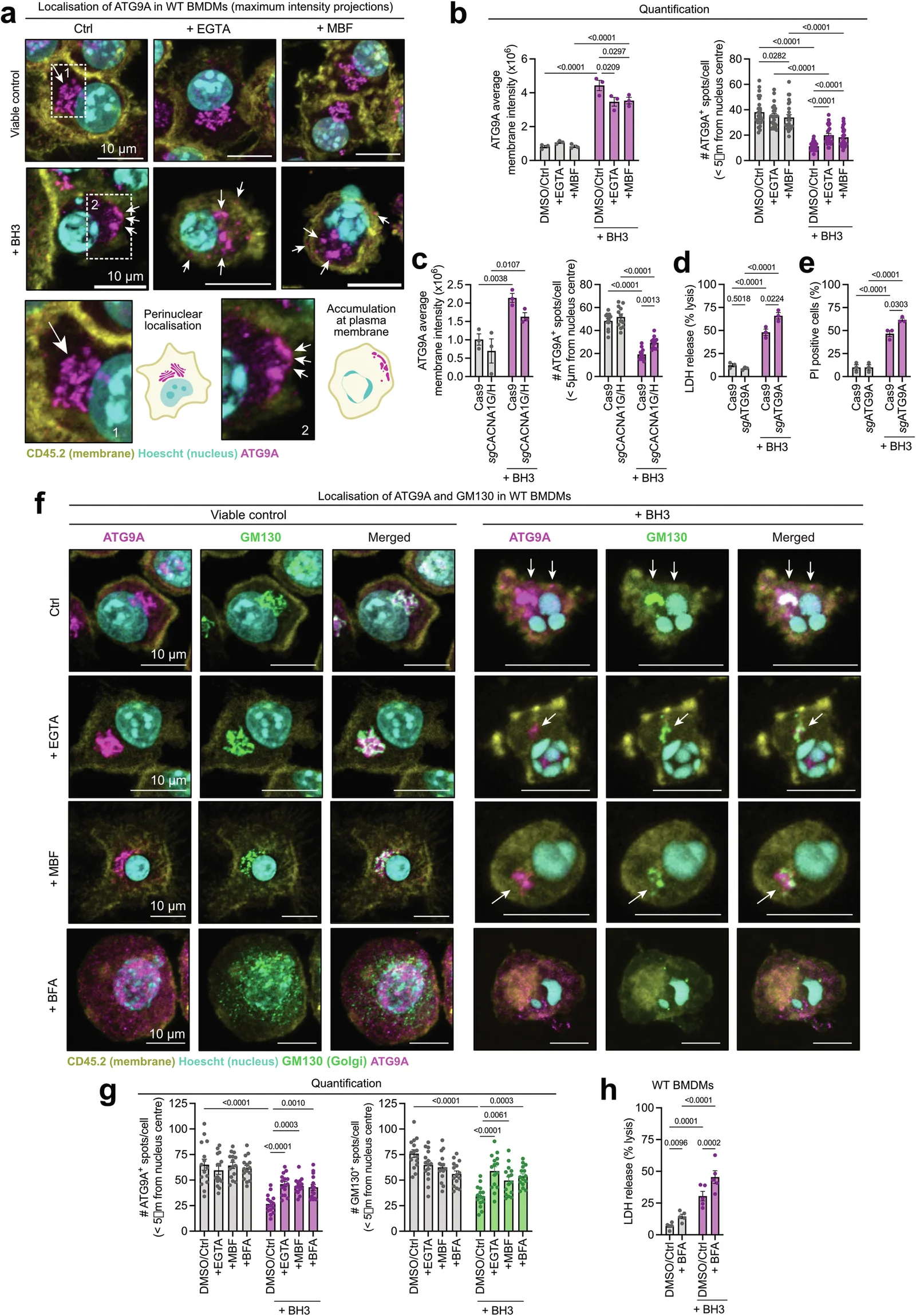
ATG9A translocation, Golgi movement, and Ca^2+^ influx through T-type channels are required for PM repair during secondary necrosis. **a** Confocal microscopy images monitoring ATG9A movement following secondary necrosis and Ca^2+^ influx blockade with EGTA (1 mM) and MBF (10 µM), and **b** associated quantitation (*n* = 3). White arrowheads denote areas of ATG9A^+^ puncta localisation (magenta) in relation to PM (yellow). ATG9A average intensity refers to median fluorescence intensity. **c** Measurement of ATG9A^+^ puncta per cell in *sg*CACNA1G/H BMDMs. **d** Measurement of membrane lysis via LDH release assay in Cas9 and *sg*ATG9A BMDMs and **e** via PI uptake. **f** Confocal microscopy observing ATG9A and GM130 movement during secondary necrosis. **g** Quantitation of ATG9A^+^ and GM130^+^ puncta per cell in relation to nucleus (cyan, Hoescht-33342) and PM (yellow, CD45.2) (*n* = 4). **h** Measurement of LDH release in BMDMs following brefeldin A treatment (5 µM) (*n* = 5). Data represent three independent experiments (*n* = 3). Error bars represent s.e.m. Statistical analyses were conducted using two-way ANOVA with Tukey’s post hoc test, *p < 0.05, **p < 0.01, ***p < 0.001; ns not significant. Scale bar = 10 μm.

Calcium entry can trigger membrane repair mechanisms upon membrane damage through the recruitment of calcium-dependent proteins [3, 15]. During lytic cell death, such as pyroptosis and necroptosis, calcium influx signals the trans-Golgi-resident ATG9A to translocate to the plasma membrane, which in turn facilitates ESCRT-dependent repair machinery [5]. To examine the role of ATG9A in limiting secondary necrosis, we monitored ATG9A following apoptosis induction by immunofluorescence microscopy. Compared to the viable control, secondary necrotic BMDMs (determined at 10 h post-apoptosis induction, based on condensed nuclei, permeabilised membrane and apoptotic cell morphology) showed increased ATG9A level at the plasma membrane proximity and less ATG9A puncta around Golgi, suggesting ATG9A translocation (Fig. 3a). The blockade of apoptotic calcium influx by ETGA or MBF significantly reversed these observations (Fig. 3a). Apoptotic BMDM deficient of T-type calcium channels also impaired ATG9A translocation to the plasma membrane (Fig. 3b). Notably, ATG9A-deficient BMDMs (Supplementary Fig. 1c) showed elevated LDH release and PI uptake without changes in apoptotic cell death (Fig. 3c, d and Supplementary Fig. 1b), highlighting the role of ATG9A in PM repair during secondary necrosis.

Given that ATG9A also functions in mobilising membrane lipids [5] and that the Golgi undergoes fragmentation during apoptosis [16], we hypothesised that the Golgi may serve as a membrane source for repair prior to secondary necrosis. Using time-lapse confocal microscopy, we observed some Golgi movement to the plasma membrane in apoptotic cells overexpressing fluorescently-tagged sialyltransferase (a proxy marker for trans-Golgi network [17] (Supplementary Fig. 2). Consistently, Golgi-resident structural protein and known marker GM130 also trafficked to the plasma membrane proximity following apoptosis induction and remained perinuclear upon blocking calcium influx, in a similar manner to ATG9A (Fig. 3e, f). Furthermore, treatment with brefeldin A, which causes the Golgi to collapse and blocks Golgi transport [18], also impaired ATG9A and GM130 translocation (Fig. 3e, f). Accordingly, brefeldin A also caused a higher level of secondary necrosis in BH3-treated BMDM (Fig. 3g).

Taken together, we demonstrated that T-type calcium channel-mediated calcium influx regulates the onset of secondary necrosis through promoting the translocation of ATG9A and delivery of Golgi membrane for membrane repair following apoptosis induction.

### T-type calcium channel-mediated calcium influx and ATG9A translocation are critical to limit unwanted secondary necrosis-associated inflammation

**Fig. 4:**
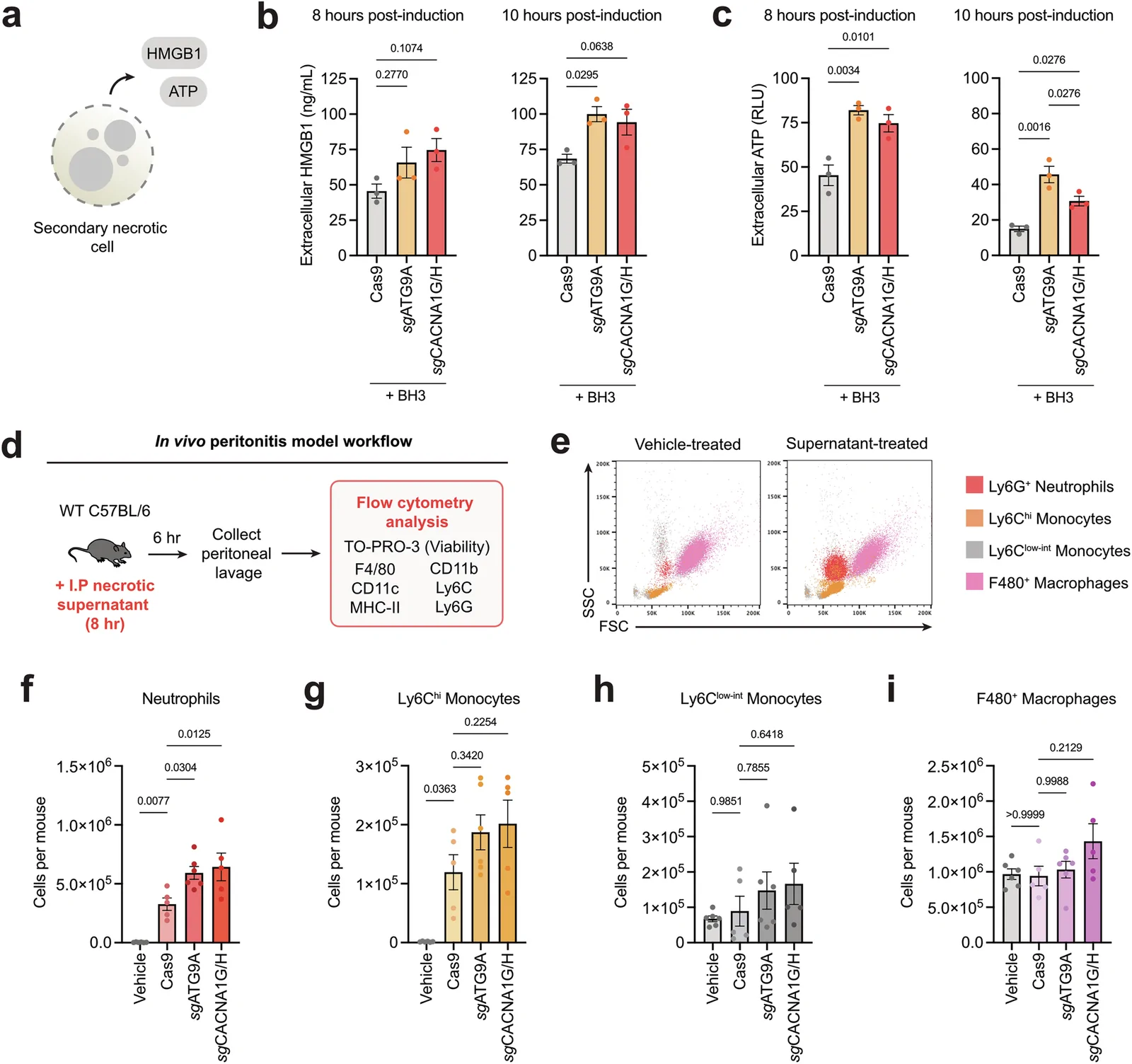
Lack of ATG9A and T-type calcium channels results in higher levels of DAMP release and immune cell recruitment in vivo. **a** Secondary necrotic cells undergo PM rupture to release DAMPs–HMGB1 and ATP. **b**, **c** Levels of HMGB1 and ATP within the supernatants of Cas9, *sg*ATG9A, and *sg*CACNA1G/H cells were measured at 8- and 10-h post-induction of apoptosis (Data representative of *n* = 3, in triplicates) **d** Schematic showing the workflow of the in vivo peritonitis model using cell-derived necrotic supernatants. **e** Changes in immune cell populations between vehicle-treated mice and secondary necrotic supernatant-treated mice. **f**–**i** Flow cytometry analysis and quantification of Ly6G^+^ neutrophils, Ly6C^hi^ monocytes, Ly6C^low-int^ monocytes, and F4/80^+^ macrophages from the peritoneum of mice treated with 8 h supernatants (*n* = 5–6). *P* values were generated using ordinary one-way ANOVA post hoc Dunnett’s multiple comparison test. Error bars represent s.e.m. *p < 0.05, ***p < 0.0001, ns, *p* > 0.05.

PMR during secondary necrosis often leads to the release of inflammatory intracellular contents, including two major DAMPs–HMGB1 and ATP (Fig. 4a). To define the functional importance of the calcium influx-dependent regulation of secondary necrosis, we found that cells deficient of T-type calcium channels or ATG9A release significantly higher levels of HMGB1 (Fig. 4b) and ATP (Fig. 4c) at 8 and 10 h post-apoptosis induction, compared to the Cas9 control. As DAMPs can trigger an inflammatory response by activating the innate immune system [19], we utilised an in vivo peritonitis model similar to the zymosan model [20] to determine if apoptotic calcium influx and ATG9A regulate secondary necrosis-associated inflammation (Fig. 4d). We collected supernatants from BMDMs at 10 h post-apoptosis induction and injected them into mouse peritoneum, then monitored the immune infiltration using flow cytometry (Fig. 4d and Supplementary Fig. 3). Compared to the media only (vehicle) control, mice treated with the supernatants from secondary necrotic BMDMs showed increased levels of infiltrated inflammatory cells, neutrophils and somewhat Ly6C^hi^ monocytes (Fig. 4e). A higher number of immune infiltrates was observed in mice treated with the supernatants from T-type calcium channel or ATG9A-dificent BMDMs, compared to Cas9 control (Fig. 4f, g). The supernatant-treated mice also had tendency to increase in Ly6C^low-int^ monocyte populations (Fig. 4h), but no changes in F4/80^+^ macrophages (Fig. 4i). Our data suggest that T-type calcium/ATG9A-mediated membrane repair during apoptosis is important to limit DAMP release and inflammatory response.

## Discussion

In this study, we uncovered an intricate mechanism of calcium influx in facilitating membrane repair to counter secondary necrosis upon apoptotic cell death. Following apoptosis induction, calcium influx, initiated by apoptosis-associated changes in membrane potential and T-type calcium channel activity, promotes the translocation of ATG9A and delivery of Golgi membrane for membrane repair in dying cells. This protective mechanism is important to regulate the onset of secondary necrosis as well as limit DAMP release and inflammatory responses.

Secondary necrosis was once considered an unregulated outcome of apoptosis [21]. However, recent studies have shown that secondary necrosis is mediated by pore-forming proteins (*i.e*., NINJ1 and GSDME) and could occur as early as GSDME is cleaved by the apoptosis executioner caspase 3 (which is activated in the early stages of apoptosis) [7, 14]. From the point of view of efferocytosis, it is logical and desirable to delay lytic and inflammatory secondary necrosis to allow sufficient time for efficient dead cell clearance, ensuring tissue homoeostasis and proper inflammation resolution [2]. To this end, apoptotic cells evidently undergo membrane depolarisation (detectable following the first sign of membrane blebbing, also a caspase-mediated event), leading to the activation of low voltage-gated T-type calcium channels for membrane repair [8]. Calcium influx has been shown to mediate membrane repair through various mechanisms, such as organelle fusion to the plasma membrane [3, 4, 22], damaged membrane budding, and removal of damaged membrane areas by neighbouring macrophages [23]. Of note, direct perturbation of the plasma membrane by physical/chemical insults and lytic cell death-associated pore-forming proteins (e.g., gasdermins, MLKL, and NINJ1) reportedly enables extracellular calcium influx through membrane lesions to trigger downstream repair processes [24]. By contrast, by using T-type calcium channels as a trigger switch, apoptotic cells can elaborately control unwanted secondary necrosis, in line with the anti-inflammatory and immunotolerant nature of apoptosis. It is worth noting that the Ca²⁺ dynamics measured by time‑lapse fluorescence imaging reflect integrated cytosolic Ca²⁺ behaviour downstream of regulated Ca²⁺ entry and do not represent direct recordings of T‑type calcium channel gating activity. Rather, our data support a model in which T‑type calcium channels provide a critical permissive switch that enables apoptosis‑associated Ca²⁺ entry required to maintain plasma membrane repair. While additional Ca²⁺ entry pathways may also contribute to shaping the overall cytosolic Ca²⁺ signal during apoptosis, their precise roles remain to be defined. Future studies employing direct electrophysiological approaches will also be important to more precisely delineate how T‑type calcium channel activity and membrane potential dynamics are coordinated during apoptosis.

The translocation of Golgi-resident ATG9A and recruitment of ATG9A-dependent repair machinery downstream of calcium influx is consistent with previous studies on lytic cell death, such as pyroptosis and necroptosis [5] suggesting a conserved repair mechanism. Furthermore, we found that the Golgi, through ATG9A translocation to the plasma membrane, may serve as a membrane source for repair prior to secondary necrosis. The donation of membrane materials for membrane repair and remodelling has been observed during cell division, exocytic patching, organelle biogenesis and autophagy [3, 16]. However, it remains to be fully elucidated in the context of the progression from apoptotic cell death to secondary necrosis. This membrane-donating mechanism may also potentially explain the unknown consequence and role of caspase-mediated Golgi fragmentation during apoptosis [25, 26].

Appropriate release of DAMPs and other intracellular contents is particularly important to ensure inflammation is only triggered when required, otherwise fuelling chronic inflammatory conditions. HMGB1 and ATP, two major DAMPs monitored in this study, are known to accumulate in the tissue to drive inflammation and immune cell recruitment [27, 28]. Elevated levels of HMGB1 and ATP released by apoptotic cells deficient in the described membrane repair machinery (ATG9A and T-type calcium channels) emphasise the importance of this regulation during apoptosis. Accordingly, in vivo administration of these DAMP-enriched supernatants also induced immune infiltration, particularly the inflammatory neutrophils and somewhat Ly6C^high^ monocytes. It is also of future interest to investigate the long-term impact of mice lacking ATG9A or T-type calcium channels in the efferocytosis-defective settings, as well as the pathogenesis of chronic inflammatory diseases and autoimmunity.

Taken together, our findings provide novel insights into the mechanism underpinning plasma membrane repair upon apoptosis. They not only highlight the elaborate regulation of the once-overlooked secondary necrosis but also offer promising strategies for therapeutic modulation of cell death and inflammation.

## Materials and methods

### Animals

C57BL/6 mice were purchased from the Walter Eliza Hall Institute of Medical Research (Kew, Melbourne, VIC, Australia). All mice were housed in specific-pathogen-free (SPF) isolators under AEC21034. All experiments were approved by the La Trobe University Animal Ethics Committee in accordance with the National Health and Medical Research Council Australia code of practice for the care and use of animals for scientific purposes.

### Cell culture

Immortalised bone marrow-derived macrophages (BMDMs) and human Jurkat T cells were obtained from WEHI and ATCC, respectively. These cells were cultured in complete DMEM or RPMI (Gibco, MA, USA) and supplemented with 10% (v/v) foetal bovine serum (FBS, Bovogen, New Zealand), streptomycin (50 mg/ml), penicillin (50 U/ml) (Life Technologies) and 0.2% MycoZap (Lonza, Switzerland).

Human-derived macrophages (hMDMs) were isolated and differentiated from PBMCs purified from human buffy coat obtained with donor consent from the Australian Red Cross Blood Service under approved ethics from the La Trobe Human Ethics Committee (Melbourne, Australia). PBMCs were isolated from buffy coat using Ficoll density gradient separation as previously reported [29]. Following, monocytes were obtained from PBMCs using CD14^+^ Microbeads and passed through an LS Microcolumn (Miltenyi Biotec) according to the manufacturer’s instructions. Macrophages were differentiated by incubating purified CD14^+^ monocytes with 1 µg/mL GM-CSF (Miltenyi Biotec, Germany) for 7 days in RPMI media supplemented with 5% hAB serum, 2 mM L-glutamine and 1 mM sodium pyruvate (Sigma-Aldrich, MO, USA).

All cells were cultured at 37 °C in 5% CO_2_-humidified atmosphere with media changes every 3–4 days.

### Induction of apoptosis and secondary necrosis

To induce apoptosis, BMDMs and hMDMs were treated with the BH3 mimetics ABT-737 (2 µM) and S63845 (10 µM) (Millenium Science, Australia), whilst human Jurkat T cells were treated with anti-Fas (0.5 µg/ml, Merck, Germany) diluted in medium supplemented with 1% BSA. To allow the progression to secondary necrosis, cells were incubated at 37 °C, 5% CO_2_ in a humidified atmosphere for 10 h. In certain experiments, cells were treated with mibefradil, EGTA, sucrose, KCl or brefeldin A during apoptosis induction (Sigma-Aldrich).

### Calcium imaging

Jurkat T cells were collected and resuspended in indicator-free DPBS at a density of 1.6 × 10⁵ cells/mL before loading with Fluo-4 AM (Thermo Fisher Scientific) at a final concentration of 2.5 μM. Cells were incubated in the dark at 37 °C for 30 min before time-lapse fluorescence imaging using a spinning disk confocal microscope (Zeiss, Germany). Images were acquired at 5 s intervals. Fluo-4 fluorescence intensity was measured from regions of interest using Fiji/ImageJ (version 2.14.0). For each cell, baseline fluorescence (F₀) was calculated as the average fluorescence intensity of the first 20 frames, and calcium-associated fluorescence changes were expressed as F/F₀. As images were acquired every 5 s, this approach was used to assess relative changes in bulk cytosolic Ca²⁺ fluorescence over time and was not designed to resolve rapid Ca²⁺ microdomains, individual Ca²⁺ transients, or T-type calcium channel gating kinetics.

### LDH release assay

The release of LDH from BMDMs, Jurkat T cells, and hMDMs was measured using the LDH Cytotoxicity Assay Kit II (Abcam, UK). After inducing secondary necrosis, samples were centrifuged at 3000×*g* for 5 min, and supernatant was collected and analysed according to the manufacturer’s instructions. Absorbance of the LDH reaction products was measured at 450 nm using a SpectraMax M5e plate reader (Molecular Devices).

### Propidium iodide (PI) uptake assay

To assess cell viability using propidium iodide (PI) staining, cells were induced to undergo secondary necrosis and stained with PI (1 mg/mL, Invitrogen) for 5–15 min at RT in the dark. The stained cells were analysed by end-point confocal imaging, and non-viable (PI-positive) cells were quantified.

### Flow cytometry

Cell viability was assessed by annexin-V binding and TO-PRO-3 uptake following induction of secondary necrosis. Levels of necrosis were determined using the BD FACSCanto III flow cytometer (BD Biosciences) in accordance with protocols described in previous studies [30]. Data analysis were conducted using FlowJo software (version 10.10.0), with the gating strategy based on the previously established method [30].

### Quantitative PCR

Total RNA was isolated from WT BMDMs using the RNeasy Mini Kit (Qiagen, Germany). cDNA was synthesised from 1 µg of RNA using the High-Capacity cDNA Reverse Transcription Kit (Applied Biosystems) according to the manufacturer’s instructions. Quantitative PCR (qPCR) was performed using TaqMan Fast Advanced Master Mix (Applied Biosystems) and TaqMan assay primers specific for CACNA1G (Mm00486572_m1), CACNA1H (Mm00445382_m1) and CACNA1I (Mm01299033_m1), with expression levels normalised to HPRT (Mm03024075_m1) as the reference gene.

### Immunoblotting

Cell pellets were obtained and lysed at 4 °C in a buffer containing 1% IGEPAL® CA-630, 10% glycerol, 1% Triton X-100, 150 mM NaCl, 20 mM HEPES, pH 7.4, with a fresh protease inhibitor tablet (Roche). The lysed samples were then subjected to SDS-PAGE and immunoblotting using the following antibodies: anti-ATG9A (1:1000; Abcam), anti-CACNA1G (1:1000; Invitrogen), anti-CACNA1H (1:500; Invitrogen) and anti-β-actin (1:5000; Sigma-Aldrich) in 1% BSA in PBST (0.1% Tween). Blots were incubated in secondary HRP-conjugated donkey anti-rabbit (1:5000, Millennium Science) in 5% milk in PBST (0.1% Tween). HRP signal was developed and detected using ECL (GE Lifesciences) and detected using the Syngene G:box gel documentation system (Syngene, MD).

### DiBAC_4_(3) staining assay

For the determination of changes in plasma membrane potential during apoptosis, DiBAC_4_(3) stock solution was diluted to 1 μM in phenol-free RPMI medium. Pluronic acid (F-127) was added to the working solution to a final concentration of 0.08% to help disperse the dye evenly. BMDMs were incubated with the DiBAC_4_(3) working solution for 30 min at 37 °C in the dark. Cells were then washed with phenol-free RPMI medium to remove excess dye. Spinning disk live imaging was performed using the 488 channel, and quantification was completed using ImageJ.

### Crosslinking assay

BMDMs were seeded at least 16 h prior to the six-well plates. After secondary necrosis induction (see figure legends for timepoints), BS [3] (bis(sulfosuccinimidyl)suberate) crosslinking agent was added to the cells (3 mM) and incubated for 7 min, RT. After, 20 mM of Tris (pH 7.5) was added to quench the reaction and incubated for 15 min. Cell pellets were harvested following centrifugation at 3000×*g* for 10 min before resuspending directly in cell lysis buffer and processed for immunoblotting (see above).

### CRISPR/Cas9 gene editing

Ca_V_3.1 and Ca_V_3.2 gene knockout in Cas9 endonuclease-expressing BMDMs was performed using a constitutive sgRNA vector CRISPR/Cas9 system similar to previously described [31]. The following oligonucleotide sequences: 5’-TCCCGTTCGAGCGAGTCAGCATGC-3’, 5’-AAACGCATGCTGACTCGCTCGAAC-3’, 5’-TCCCTGCCAGCGTGCCCTCGGTCA -3’, 5’-AAACTGACCGAGGGCACGCTGGCA -3’ that correspond to Ca_V_3.1 and Ca_V_3.2 gRNA, respectively and were designed using the primer design tool on the Synthego website (design.synthego.com) [32]. annealed and ligated separately in the constitutive pFgh1tUTG-CFP plasmid, digested with the *Bsm*BI restriction enzyme. Human embryonic kidney cells (HEK 293T) were transfected with pFgh1tUTG‑CFP‑CaV3.1 and pFgh1tUTG‑CFP‑Ca_V_3.2, pCMV and human lentiviral receptor. The lentiviral supernatant collected from transfected HEK 293T cells was filtered, added with 10 µg/mL polybrene and used to spin transduce Cas9-expressing Jurkat T cells (1500×*g*, 37 °C, 45 min). Cas9‑expressing BMDMs were incubated for 6 h to allow transduction to take place, and the media was changed at the end of the incubation period. Cas9-expressing BMDMs were then incubated for 72 h in complete DMEM. Bulk sorting was performed using FACS Aria II (BD Biosciences) to identify mCherry and CFP-expressing cells.

### Immunofluorescence confocal microscopy

BMDMs were seeded at 7.5 × 10^4^ cells/well one day prior. Media was removed, and cells were washed gently three times with PBS before staining with CD45.2 PE (1:100, BD Biosciences) in 1% BSA/DMEM for 5 min at RT in the dark. Cells were then washed with PBS, and apoptosis was induced. Next, cells were fixed and permeabilised with 2% paraformaldehyde/PBS for 10 min at RT, and 0.5% saponin/PBS for 20 min at RT. Cells were then blocked with 1% BSA for 30 min RT, then incubated overnight with anti-ATG9A (1:100) at 4 °C. The following day, samples were incubated using secondary antibody donkey anti-rabbit IgG Alexa Fluor-647 (ab150075, Abcam) for 1 h, RT. Samples were washed, then incubated with Hoescht-33342 (0.1 µg/mL, Invitrogen) for 5 min before performing end-point confocal imaging using the LSM 900 Confocal Microscope using the oil-immersion 63x/1.46 objective lens (Zeiss, Germany). Pixel size was 0.198 μm and voxel depth for z-stacks was maintained at 0.5–1 μm.

For experiments observing GM130 (an established Golgi marker) movement, samples were incubated with anti-GM130 Alexa Fluor-488 (1:50, BD Pharminogen) for 1 h, RT after overnight incubation with anti-ATG9A.

Analysis of ATG9A and GM130 localisation was conducted using z-stacks and spot and surface analysis integrated in Imaris (version 9). Spots and surfaces determined to be close to the nucleus were defined as being <5 µm from the centre. For representation in the manuscript, images were processed into maximum-intensity projections.

### Preparation of necrotic supernatant

Cas9, *sg*ATG9A or *sg*CACNA1G/H cells were seeded at 3.6 × 10^6^ cells/10 cm plate (6.25 × 10^4^/cm^2^) at least 16 h prior to treatment. Cells were induced to undergo secondary necrosis via treatment with a BH3 mimetic cocktail made up in 3 mL of 1% BSA/DMEM media before incubating at 37 °C, 5% CO_2_. At 8 and 10 h post-induction, the remaining cell supernatant was harvested and differentially centrifuged at 4000×*g* for 15 min to remove any remaining cellular debris. The remaining supernatant was harvested and snap-frozen in liquid nitrogen before storage at −80 °C. Supernatants underwent quality control via LDH release assay prior to use.

### HMGB1 release assay

HMGB1 release assay was performed using Lumit™ HMGB1 (Human/Mouse) Immunoassay (Promega) following the manufacturer’s instructions. Briefly, snap-frozen supernatants were thawed on ice, and aliquoted into an opaque 96-well plate. Next, anti-HMGB1 mAb-SmBiT and anti-HMGB1-LgBiT were added at 1:50 and incubated for 60 min, covered at RT. To detect HMGB1 levels, a 1:20 dilution of the detection substrate/reagent B mixture was dispensed into each well and incubated for 5 min before reading luminescence using CLARIOstar.

### ATP release assay

ATP release assay was performed using the RealTime-Glo^TM^ Extracellular ATP kit (Promega) using the manufacturer’s instructions. Briefly, Cas9, *sg*ATG9A, *sg*CACNA1G/H cells were seeded at 2 × 10^4^ cells/well in triplicates in 96-well white, opaque plate at least 16 h prior to assay. Cells were gently washed with 1x PBS before adding assay reagent and treatment media diluted to 1x in the plate. Plate was allowed to equilibrate to 37 °C in CLARIOstar. Readings were taken every 10 min for 12 h.

### Peritonitis model

C57BL/6 mice (male and female) were housed and used under La Trobe University Animal Ethics Committee approval (AEC21034). Snap-frozen necrotic supernatants were thawed on ice and intraperitoneally injected into WT C57BL/6 mice at 300 µL per mouse. After 6 h post injection, mice were euthanised via CO_2_ asphyxiation, and peritoneal lavages were collected by flushing with 5 mL PBS. The collected cells were spun at 500×*g* for 10 min and resuspended in FACS buffer (PBS/2 mM EDTA/2% FCS). Cells were first blocked with CD16/CD32 FC block (BD Bioscience #553142) before staining with TO-PRO-3^TM^ (Invitrogen, Cat #T3605), F4/80 BUV496 (BD Bioscience, #750644), CD11C PE (BD Bioscience, #557401), I-A/I-E FITC (BD Bioscience, #553623), CD11B PeCy7 (Invitrogen, #25-0112-82), Ly6C BV421 (BD Bioscience, #562727), Ly6G Pe/Dazzle594 (BioLegend, #127648), and the migration of immune cells was assessed by flow cytometry using the FACS A3 Symphony. Results were analysed using FlowJo (version 10.10.0).

### Statistics

All statistical analyses were performed using GraphPad Prism v10 (San Diego, CA). Data were represented as mean ± SEM unless otherwise stated. Where appropriate, one-way or two-way ANOVA analysis, with a Tukey’s or Dunnett’s post hoc test was applied. A *p* value of <0.05 was considered significant.

## Supplementary information

Supplementary Figure Titles

Supplementary Figure 1

Supplementary Figure 2

Supplementary Figure 3

Uncropped Western Blots

## Data Availability

The data generated in this study are presented within this article and supplementary information. Further information and data are available upon request from the corresponding authors, kha.phan@latrobe.edu.au and i.poon@latrobe.edu.au.
