# Supplementary material for: Calcium influx through T-type calcium channels regulates secondary necrosis and inflammation through recruitment of ATG9A-mediated membrane repair
Source: Cell Death Dis. 2026 Jul 8;17(1):849. doi: 10.1038/s41419-026-09070-9 (PMC13631255; doi:10.1038/s41419-026-09070-9)
Supplement: Supplementary file 1 [file 41419_2026_9070_MOESM1_ESM.docx]

**Supplementary Figure 1. sgCACNA1G/H and sgATG9A BMDMs undergo apoptosis and secondary necrosis. a.** Loss of CACNA1G and CACNA1H in *sg*CACNA1G/H targeted BMDMs compared to Cas9 controls. **b.** Loss of ATG9A expression in CRISPR/Cas9 targeted *sg*ATG9A BMDMs compared Cas9 controls. **c.** Caspase 3/7 activity is the same across Cas9, *sg*ATG9A, and *sg*CACNA1G/H BMDMs. d. LDH release assay of BMDMs at 10 h post-apoptosis with necrostatin-1 or QVD treatment, suggesting BMDM membrane lysis is apoptosis-driven, independently of necroptosis. P-values were generated using ordinary two-way ANOVA post-hoc Tukey’s multiple comparison’s test. Error bars represent s.e.m. *P<0.05, *** P<0.0001, NS = P>0.05. All data representative of 2 independent experiments.

**Supplementary Figure 2. Movement of golgi-specific component Sialyltransferase in Clover-SiT-N-15 overexpressing A431 cells.** Scale bar = 10 μm.

**Supplementary Figure 3. Gating strategy for intraperitoneal immune cells.**
